# Extreme drought shapes the gut microbiota composition and function of common cranes (*Grus grus*) wintering in Poyang Lake

**DOI:** 10.3389/fmicb.2024.1489906

**Published:** 2024-11-20

**Authors:** Chaoyang Wang, Chao Zhang, Hao Cai, Yunlong Zhu, Jiwan Sun, Wen Liu, Zhenyu Wang, Yankuo Li

**Affiliations:** ^1^College of Life Sciences, Jiangxi Normal University, Nanchang, China; ^2^State Key Laboratory of Integrated Management of Pest Insects and Rodents, Institute of Zoology, Chinese Academy of Sciences, Beijing, China; ^3^Office of Poyang Lake Water Control Project Construction of Jiangxi Province, Nanchang, China

**Keywords:** gut microbiota, common crane, extreme drought, water-level fluctuations, Poyang Lake

## Abstract

**Introduction:**

Extreme weather events driven by climate change profoundly affect migratory birds by altering their habitats, food sources, and migration routes. While gut microbiota is believed to play a role in helping birds adapt to environmental changes, research on how extreme weather impacts their gut microbiota and how these microbial communities respond to such conditions has been limited.

**Methods:**

16S rRNA gene sequencing was utilized to investigate the gut microbiota of common cranes (*Grus grus*) wintering at Poyang Lake from 2020 to 2023, with a particular focus on their response to extreme drought conditions on both inter-annual and monthly timescales.

**Results:**

The results revealed that extreme drought conditions substantially impact gut microbiota, with inter-annual water-level fluctuations exerting a more pronounced impact on microbial community structure than that of inter-monthly fluctuations. Notably, a significant decline in bacterial diversity within the gut microbiota of common cranes was observed in the extreme drought year of 2022 compared with other years. Monthly observations indicated a gradual increase in gut microbial diversity, coinciding with relatively minor water-level changes. Key taxa that responded to drought included the Enterobacteriaceae family and *Bifidobacterium* and *Lactobacillus* species. Additionally, functional genes related to carbohydrate metabolism, the phosphotransferase system, and the two-component systems were significantly enriched during the extreme drought year. These functions may represent adaptive mechanisms by which the gut microbiota of common cranes respond to drought stress.

**Discussion:**

This research provides novel insights into the temporal variability of gut microbiota in wintering waterbirds, underscoring the significant impact of climatic fluctuations on microbial communities. The findings highlight the importance of understanding the ecological and functional responses of gut microbiota to extreme weather events, which is crucial for the conservation and management of migratory bird populations in the face of climate change.

## 1 Introduction

The global climate crisis has intensified dramatically in recent years, as evidenced by rising global temperatures, altered precipitation patterns, and an increased frequency of extreme weather events (Ghanbari et al., 2023). These climatic changes have far-reaching impacts on global biodiversity and ecosystem functionality, leading to declining survival rates of numerous species, heightened prevalence of pathogens, and an elevated risk of species extinctions (Upadhyay, 2020; Williams et al., 2023). Traditional predictive models of wild animal responses to climate change have primarily focused on physiology, behavior, population genetic variation, dispersal ability, and habitat requirements (Garcia-Costoya et al., 2023; Neel et al., 2021). However, the role of host-associated microbial communities, which inhabit nearly all organisms, has been relatively underexplored. Emerging research underscores that climate-induced changes in these microbial communities can profoundly affect host resilience to environmental stressors (Sepulveda and Moeller, 2020; Williams et al., 2020). Such microbial shifts may either amplify the negative effects of climate change on hosts or enhance host adaptability and survival. Consequently, predicting the impacts of climate change on animals necessitates a comprehensive understanding of how these changes will affect both the host and its associated microbial counterparts (Kolodny and Schulenburg, 2020).

For wild animals, the gut microbiota is influenced by a combination of intrinsic factors, such as genetics (Groussin et al., 2020), age (Tian et al., 2020), sex (Li Y. et al., 2021), and behavior (Johnson et al., 2022), as well as extrinsic factors such as diet (Mason et al., 2020) and environmental (Tang et al., 2023) conditions. While a connection between host genetics and microbiomes in mammals has been well-documented, investigations into avian microbiomes suggest these genetic links may be less pronounced (Song et al., 2020). With their remarkable flying capabilities and complex life histories, birds harbor gut microbiomes that are particularly sensitive to environmental changes (Kohl, 2012). Environmental stressors, such as temperature fluctuations, seasonal pattern changes, precipitation variability, and extreme weather events, interact intricately and profoundly influence the microbial ecosystems within bird guts (Williams et al., 2023). These interactions are critical to the birds’ adaptive mechanisms. Furthermore, understanding avian responses to environmental challenges is essential for assessing their resilience and adaptability to the increasing threats posed by climate change.

The Poyang Lake wetland is renowned for its remarkable biodiversity and stands as one of the largest wintering grounds for migratory birds globally. A primary factor contributing to the high biodiversity in Poyang Lake is the significant fluctuations in water levels over both short- (weeks to months) and long-term (years) periods (Andreoli et al., 2007). These fluctuations foster extensive wetland complexity in Poyang Lake, providing vital wintering habitats for thousands of migratory birds (Liu et al., 2011). Water-level changes not only reshape the landscape of waterbird habitats, but also alter the availability and composition of food sources for herbivorous wintering waterbirds. In recent years, the hydrological conditions at Poyang Lake have fluctuated rapidly, driven mostly by climatic change and human activities. Notable events include the summer flooding in 2020 (Zhang et al., 2022), extreme drought in 2022 (Gui et al., 2024), and the drought during the typically high-water period in 2023. These events present an excellent opportunity to explore how the gut microbiota of wintering birds at Poyang Lake responds to hydrological fluctuations primarily caused by climate change. Understanding these microbial responses is crucial for assessing the adaptive strategies of migratory birds and for developing effective conservation measures in the face of ongoing environmental changes.

Cranes (Gruiformes, Gruidae) represent one of the most endangered groups of birds. Poyang Lake serves as a critical wintering habitat, supporting approximately 98% of the global population of Siberian cranes (*Leucogeranus leucogeranus*), most of the Chinese population of white-naped cranes (*Grus vipio*), one-third of hooded cranes (*G. monacha*), and half of the common cranes (*G. grus*) (Li et al., 2019). The overwintering environment at Poyang Lake profoundly affects the cranes’ physical health, influencing their survival rates, migration schedules, and reproductive success in the subsequent year (Li et al., 2020). To maintain optimal health during the overwintering period, cranes have evolved several adaptive behavioral strategies, such as shifting habitats and modifying foraging strategies (Jiang et al., 2015).

This study aimed to investigate the temporal changes in the gut microbiota of common cranes during their wintering period at Poyang Lake, considering both large- (inter-annual) and small-scale (inter-month) temporal variations. By employing 16S rRNA gene sequencing, we described and compared the gut microbiota composition of common cranes across these temporal scales. This comparative analysis sought to uncover the adaptability and resilience of gut microbiota in response to ecological and physiological changes induced by water-level fluctuations at Poyang Lake. Our findings aimed to enhance the understanding of the interconnections between the gut microbiota of wintering common cranes and temporal environmental changes, providing valuable insights into the adaptive strategies of migratory birds in their wintering habitats.

## 2 Materials and methods

### 2.1 Study areas and sample collection

The study was conducted at Poyang Lake, situated in northern Jiangxi Province, China (28°11′–29°51′N, 115°49′–116°46′E). As the largest freshwater lake in China, Poyang Lake is integral to the region’s hydrological processes and holds significant international ecological importance. The lake is located within a subtropical humid monsoon climate zone, which experiences hot and rainy summers and warm and humid winters. The area receives annual precipitation ranging from 1,350 to 2,150 mm, and the average temperature fluctuates between 16.5 and 17.8°C (Wang S. Y. et al., 2021). The lake’s water levels undergo significant seasonal variations, alternating between flood and dry phases, fostering various wetland ecosystems rich in biodiversity. These fluctuating conditions provide crucial habitats that support an abundance of species, including hundreds of thousands of waterbirds that migrate to the area annually. Poyang Lake is particularly notable as a key wintering site for endangered species such as the Siberian, common, and hooded cranes (Li et al., 2020). The intersection of its unique climatic and hydrological characteristics highlights Poyang Lake’s vital role as a sanctuary for avian biodiversity, emphasizing its importance in global conservation efforts.

**Figure 1:**
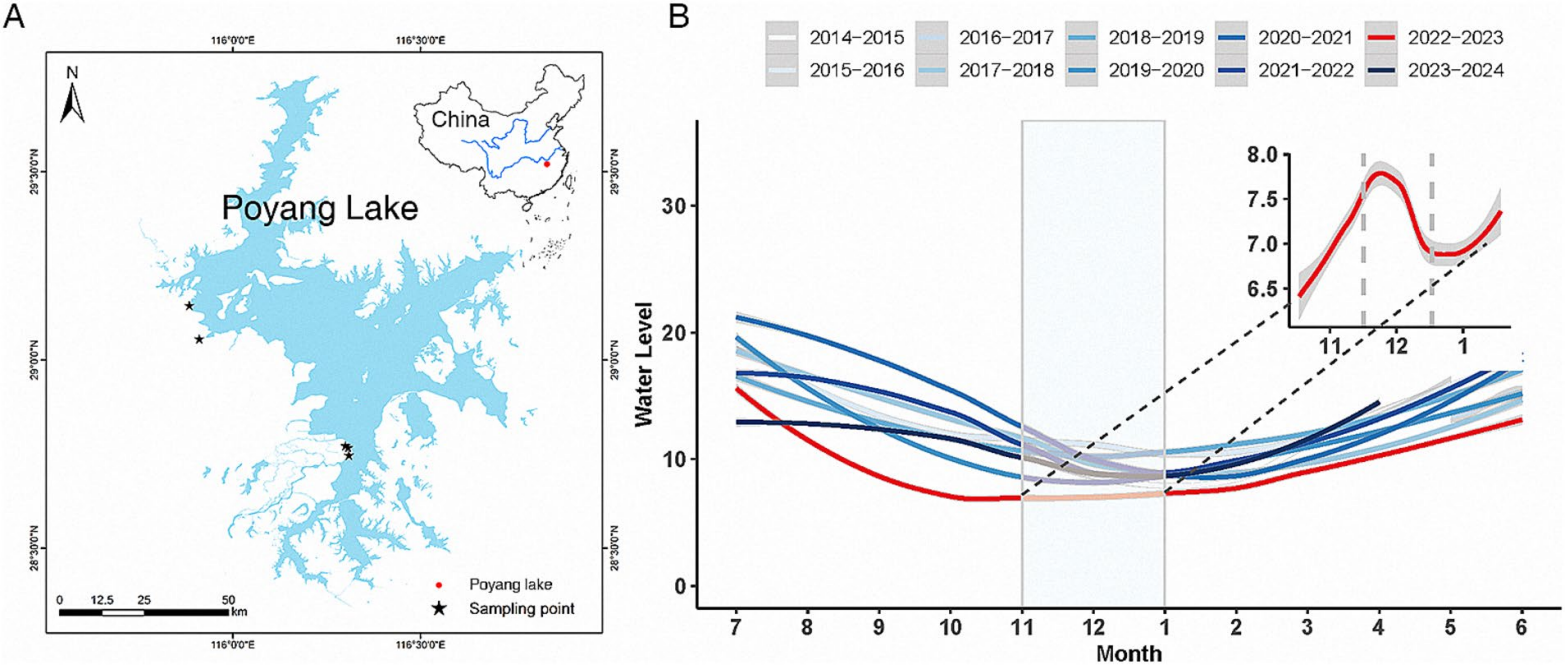
Common crane feces sampling sites at Poyang Lake. The inset in the top right corner illustrates the geographical location of Poyang Lake within China **(A)**. The main panel **(B)** depicts the trend of water level changes at Poyang Lake over the past decade, along with a detailed view of water-level fluctuations during November–December 2022 in the upper right corner.

In this study, fresh fecal samples from common cranes were systematically collected from paddy field habitats around Poyang Lake (Figure 1A). The collection was conducted across different time periods, yielding a total of 58 samples: 12 in November 2020 (2020 group), 10 in November 2022 (2022/Nov group), 12 in December 2022 (Dec group), 12 in January 2023 (Jan group), and 12 in November 2023 (2023 group). To ensure accurate sampling, we initially used binoculars to observe large flocks of common cranes during their feeding activities. We specifically targeted areas where cranes were feeding in significant numbers, ensuring that no other cranes or waterfowl were present within a 50 m radius during our observations. After the cranes had completed feeding and vacated the site, we quickly moved to the area to collect samples. Feeding sites were identified based on visible crane footprints or feeding pits, and we took care to collect each fecal sample at least 5 m apart to prevent cross-contamination (Zhang et al., 2012). Additionally, we collected more than 20 fecal samples at each sampling time point and randomly selected 12 samples for sequencing. This approach may help mitigate the risk of repeated sampling to some extent. Samples were collected using sterile disposable gloves and tweezers, with any portions in contact with the ground being discarded. The samples were then immediately placed into sterile 50 mL centrifuge tubes, properly labeled, and transported back to the laboratory on liquid nitrogen. Upon arrival, they were stored in an ultra-low temperature freezer at −80°C to preserve their integrity for further analysis.

### 2.2 Hydrological data

Poyang Lake serves as a crucial habitat and food source for wintering migratory birds, with these resources closely linked to the lake’s fluctuating water-levels (Xia et al., 2010). To investigate how the gut microbiota of common cranes responds to drought, we utilized real-time water-level data from Poyang Lake, which was obtained from the official Jiangxi Provincial Water Resources Department website.^1^ Water-level readings were recorded daily at 9:00 and 15:00 from multiple hydrological stations around the lake. Our analysis focused on data from the Xingzi Station, where monthly average water levels were calculated to assess long-term trends. To identify broader patterns, we examined historical monthly average water levels from 2014 to 2023 (Figure 1B) and analyzed daily average water levels during the specific months when samples were collected. Beginning in July 2022, extreme weather conditions characterized by high temperatures and low rainfall, along with reduced water flow from the upper reaches of the Yangtze River, led to an early onset of the dry season in Poyang Lake and unprecedented drought conditions not seen in decades. According to a red drought warning issued by the Jiangxi Provincial Hydrological Monitoring Center on September 27, 2022, Jiangxi province experienced sustained drought with rainfall levels 60% below the historical average since July. Consequently, the inflow from the five major rivers feeding Poyang Lake was reduced by nearly 60%, with the water level at Xingzi Station reaching 6.97 m, 0.14 m below the historical minimum. Thus, 2022 was an unequivocally dry year for the Poyang Lake region. In contrast to the severe drought in 2022, water levels in Poyang Lake during the dry seasons of 2020 and 2023 fluctuated closer to the historical average (Figure 1B; Supplementary Figure S1). During the drought in 2022, water levels gradually rose from November 1 to December 7, peaking at 8.41 m before declining to 6.78 m by January 7, after which levels began to rise again. These interannual and intra-annual fluctuations in water levels likely influence the environmental conditions that affect the gut microbiota of common cranes, highlighting the dynamic interactions between hydrological factors and avian health.

### 2.3 DNA extraction and sequencing

Bacterial DNA was extracted from the collected samples using a QIAamp DNA Stool Mini Kit (QIAGEN, Hilden, Germany), following the manufacturer’s recommended procedure. To target the V3–V4 hypervariable region of the 16S rRNA gene, we employed specific primers with barcodes: 343F (5′-TACGGRAGGCAGCAG-3′) and 798R (5′-AGGGTATCTAATCCT-3′). PCR amplification was conducted in a total reaction volume of 25 μL, which included 8.5 μL of ddH_2_O, 12.5 μL of 2× TaKaRa Taq polymerase (TaKaRa Bio Inc., Shiga, Japan), 1 μL of each primer, and 2 μL of the DNA template. The thermal cycling conditions were as follows: an initial denaturation at 95°C for 5 min, followed by 30 cycles of denaturation at 95°C for 30 s, annealing at 55°C for 60 s, and extension at 72°C for 60 s, with a final extension step at 72°C for 5 min. To verify successful amplification, 2 μL of the PCR products were analyzed via gel electrophoresis using a 1.5% agarose gel, run at 100 V for 60 min. Following confirmation, the PCR products were purified using an Agencourt AMPure XP kit (Beckman Coulter, Brea, CA, United States), adhering to the manufacturer’s guidelines. The purified PCR products was sequenced on an Illumina HiSeq PE150 platform (Illumina, San Diego, CA, United States), according to the established protocol, ensuring high-quality sequencing data for subsequent analysis.

### 2.4 Bioinformatics analysis

Raw sequence reads underwent quality filtering and truncation before being processed to infer amplicon sequence variants (ASVs) using the DADA2 pipeline (Callahan et al., 2016). Post quality control, the paired reads were merged, and any chimeric sequences were removed, allowing for the clustering of reads into distinct ASVs. Taxonomic classification for each ASV was then performed using a naive Bayes classifier, which had been trained on the SILVA database (v138). To further analyze the data, a phylogenetic tree of the ASV sequences was generated using FastTree, based on multiple sequence alignments produced using the DECIPHER package (Wright, 2016). The resulting ASV abundance table, along with taxonomic classifications, the phylogenetic tree, and associated metadata, were compiled into a phyloseq object to facilitate statistical analysis (Mcmurdie and Holmes, 2013). Subsequently, alpha diversity metrics, including Shannon diversity, Observed species count, and Chao1 richness estimates, were calculated using the *estimate_richness* function within the phyloseq package after rarefying the data to an equal sequencing depth across samples. To explore the potential functional roles of the identified ASVs, functional predictions were made using PICRUSt2 (Douglas et al., 2020), providing insights into the ecological functions of the microbial communities.

### 2.5 Statistical analysis

Non-metric multidimensional scaling (NMDS) based on Bray–Curtis dissimilarity was employed to assess the variation in microbial composition across different time periods. To statistically evaluate the differences in microbiota composition, permutational multivariate analysis of variance (PERMANOVA) was conducted using the “adonis2” function from the vegan package in R (R Foundation for Statistical Computing, Vienna, Austria) (Martino et al., 2019). The Kruskal–Wallis test was employed to analyze differences in alpha diversity among the groups. To identify specific group differences, *post hoc* pairwise comparisons were conducted using the Steel-Dwass method. The community composition at various taxonomic levels was visualized using alluvial plots and heatmaps, constructed using the “ggalluvial” package in R, offering a clear depiction of shifts in microbial populations over time. To further explore the dynamic changes in microbial communities between two temporal scales, the Wilcoxon signed-rank test was utilized to compare microbial community distances, allowing for a more detailed understanding of temporal variability in microbial composition. All analyses were conducted using the statistical software R v.4.2.3 (Brunson, 2020).

## 3 Results

### 3.1 Impact of extreme drought on gut microbiota diversity and structure in common cranes

A total of 58 fecal samples were collected during the wintering periods of 2020, 2022, and 2023. After quality control and denoising, 3,251,642 valid reads were obtained, with an average of 56,062 sequences per sample. Across all samples, 12,077 ASVs were identified. ASVs not annotated to the phylum level and those present in fewer than 5% of the samples were excluded from further analysis. Additionally, only ASVs with a read count exceeding 200 in individual samples were retained, resulting in a final total of 1,504 ASVs. The rarefaction curves reached a plateau, indicating that the sequencing depth was sufficient to capture most of the microbial diversity present in the samples (Supplementary Figure S2). On an inter-annual basis, 138 ASVs (9.8% of the total ASVs identified over 3 years) were shared, with 2022 having the fewest unique ASVs at 60 (4.3%). On an inter-monthly basis, 201 ASVs (24.9% of total ASVs) were shared across three successive months, with November exhibiting the fewest unique ASVs, totaling 64 (7.9%) (Supplementary Figure S3).

**Figure 2:**
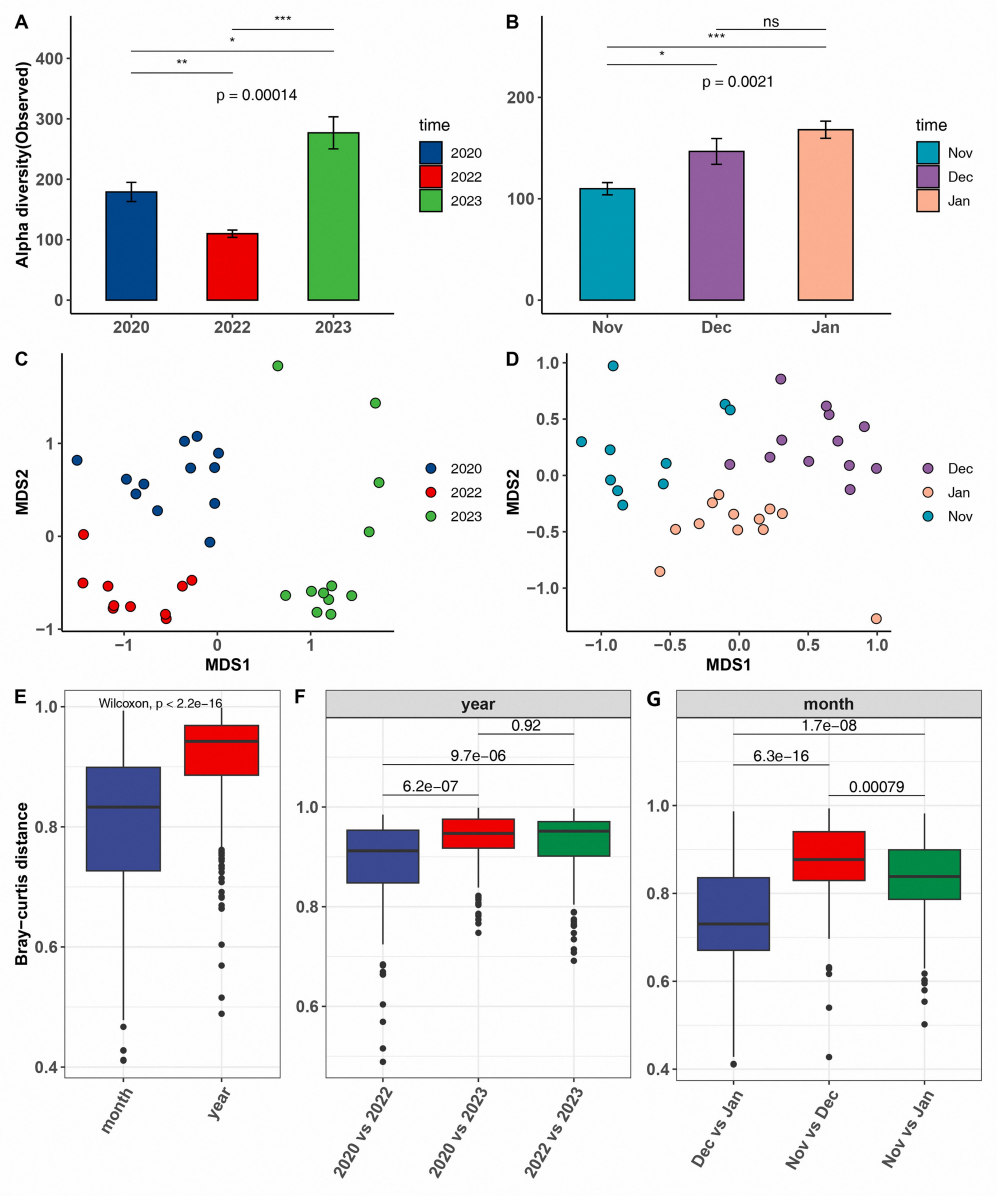
Comparison of alpha diversity, measured by the number of observed amplicon sequence variants, in common cranes across both inter-annual **(A)** and inter-monthly temporal scales **(B)**. Non-metric multidimensional scaling (NMDS) analysis based on Bray–Curtis dissimilarity matrices across both inter-annual **(C)** and inter-monthly temporal scales **(D)**. Comparison of the magnitude of change in microbial community distances across the two temporal scales using the Wilcoxon signed-rank test (**E–G**).

The alpha diversity analysis revealed a significant decline in the gut microbiota diversity of common cranes during the extreme drought year of 2022 compared with that in other years. This trend was consistent across various alpha diversity metrics, including observed (Figures 2A,B), Chao1, Shannon, and Faith’s PD (Supplementary Figure S4). In contrast, the diversity in the wetter year of 2023 was markedly higher than in other years. On a monthly scale, from November 2022 to January 2023, the alpha diversity of common cranes microbiota exhibited an increasing trend, corresponding with rising water levels during these periods.

PERMANOVA analysis based on Bray–Curtis distances revealed significant differences in microbial community structure at both annual and monthly scales (*R*^2^ = 0.4416, *p* = 0.001 for inter-annual; *R*^2^ = 0.3695, *p* = 0.001 for inter-monthly). These differences are clearly illustrated in the NMDS plot (Figures 2C,D). To further investigate these variations, we compared the Bray–Curtis distances between inter-annual and inter-monthly datasets, as well as the pairwise differences between individual years and months. As anticipated, the microbial community differences were more pronounced on an annual scale than on a monthly scale (Figure 2E). Among the different years, the microbial community composition in 2023 was significantly distinct from that in other years. On a monthly scale, November 2022 exhibited the greatest difference in microbial community composition compared with that in the subsequent months in 2023 (Figures 2F,G).

### 3.2 Identification of key microbial taxa responding to extreme drought: enrichment and depletion patterns

**Figure 3:**
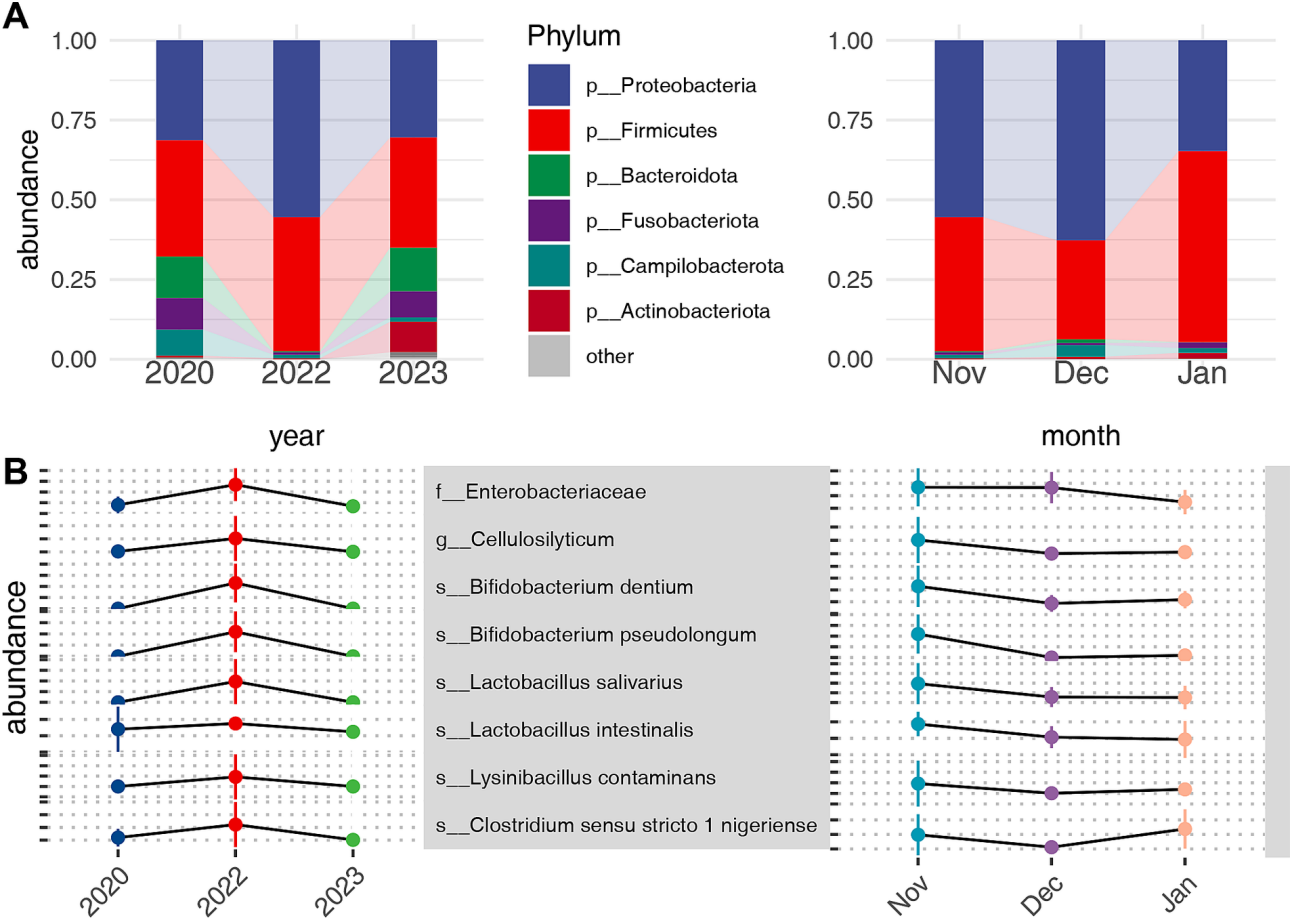
Relative abundance of bacterial phyla in the microbiota of common cranes across different temporal scales **(A)**. Bacteria with the highest relative abundance in 2022 at the family, genus, and species levels within inter-annual temporal scales and their trends over inter-monthly temporal scales **(B)**.

The gut microbiota of common cranes comprised 16 phyla, 24 classes, 75 orders, 139 families, 294 genera, and 171 species. At the phylum level, the highest average relative abundance was observed for Proteobacteria (42.45 ± 25.94%), followed by Firmicutes (40.79 ± 22.54%), Bacteroidota (5.8 ± 12.21%), Fusobacteriota (4.3 ± 13.56%), Campilobacterota (3.25 ± 4.91%), and Actinobacteriota (2.70 ± 6.26%). These six phyla collectively accounted for 99.39% of the total reads, with the remaining 0.61% distributed among 10 other phyla (Figure 3A) (Supplementary Table S1). At the genus level, 12 genera, including *Enterobacter* (19.0 ± 24.0%), *Lactobacillus* (11.59 ± 16.16%), *Escherichia-Shigella* (8.57 ± 18.43%), and *Clostridium sensu stricto 1* (5.28 ± 8.25%), exhibited an average relative abundance exceeding 2% (Supplementary Table S2).

To identify microbial taxa responding to extreme drought, we employed the Kruskal–Wallis test at various taxonomic levels, followed by pairwise post-hoc testing using the Steel-Dwass method. This approach allowed us to examine microbial community differences across both annual and monthly scales. We specifically focused on notably enriched or depleted taxa in 2022 and found no significant differences between 2020 and 2023. According to our criteria, we identified key taxa enriched under drought conditions. At the family level, Enterobacteriaceae showed significant enrichment. At the genus level, *Cellulosilyticum* was notably increased, while at the species level, taxa primarily from *Bifidobacterium* and *Lactobacillus* were enriched (Figure 3B). Conversely, several taxa exhibited significant depletion during the extreme drought. These included two phyla, Bacteroidota and Desulfobacterota, as well as various families, genera, and species, including *Bacteroides vulgatus*, *Bacteroides uniformis*, and *Faecalibacterium prausnitzii* (Supplementary Figure S5). Notably, these taxa exhibited opposite trends on an inter-annual basis compared with their response to alleviated water levels on an inter-monthly scale.

### 3.3 Functional mechanisms and microbial response patterns to extreme drought

**Figure 4:**
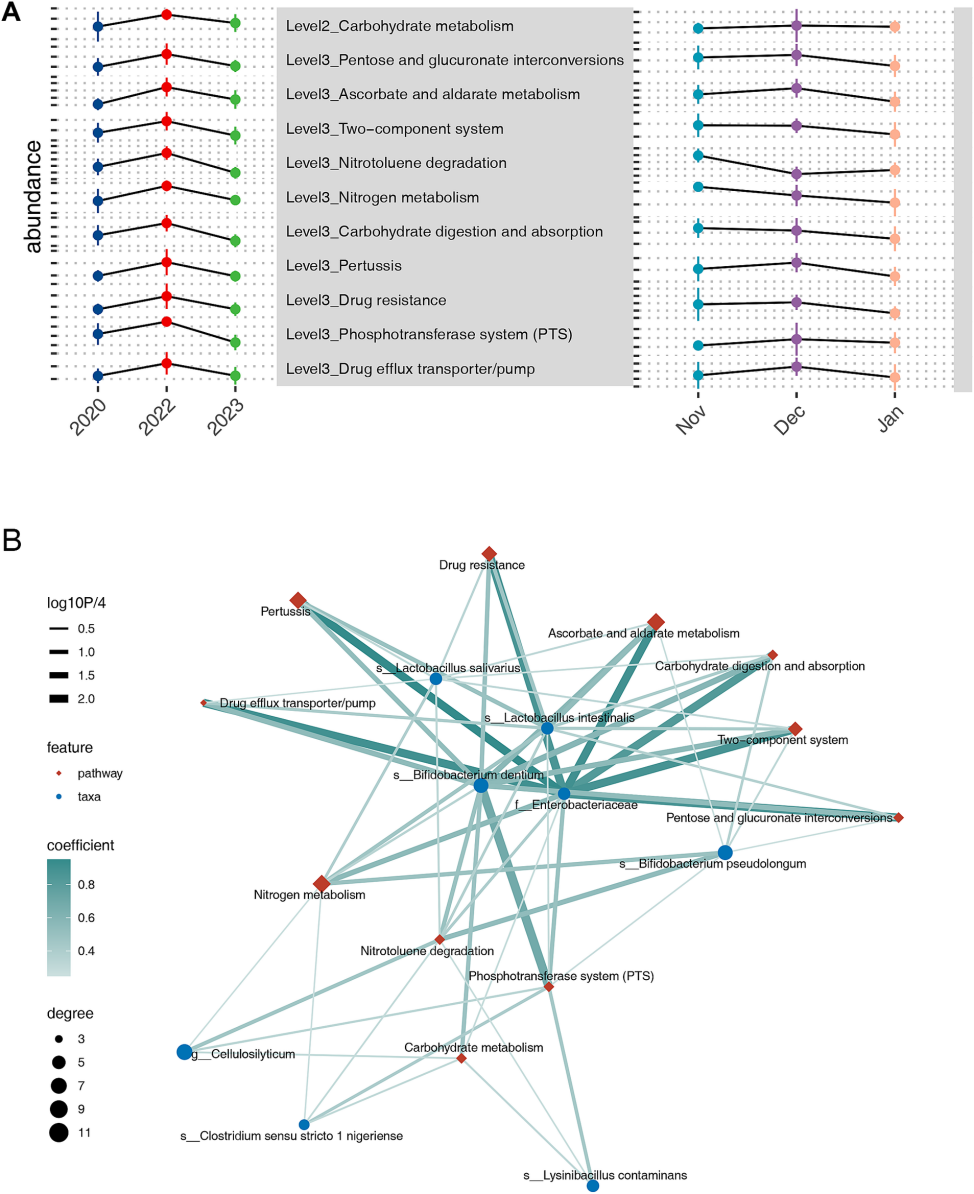
Kyoto Encyclopedia of Genes and Genomes pathway analyses across inter-annual temporal scales, highlighting the pathways with the highest abundances in 2022 at levels 2 and levels 3, along with their abundance trends over inter-monthly temporal scales **(A)**. Correlation network analysis illustrating the relationship between bacteria with higher abundance in 2022 and functional pathways over inter-annual temporal scales **(B)**.

We explored the potential mechanisms by which the gut microbiota responds to drought at the functional level. Using PICRUSt2, we predicted Kyoto Encyclopedia of Genes and Genomes (KEGG) orthologies and aggregated their abundances to determine the prevalence of KEGG pathways at various levels (Supplementary Figure S6). At level 2 of KEGG pathways, we observed that carbohydrate metabolism was notably enriched in the extreme drought year 2022. At levels 3, 14 pathways were enriched during the drought year, including several associated with environmental stress responses, such as the two-component system, nitrogen metabolism, phosphotransferase system (PTS), and drug efflux transporter/pump (Figure 4A). Conversely, numerous other pathways or functions were diminished compared with the other years (Supplementary Figure S7).

To further analyze the relationships between drought-responsive functions and microbial taxa, we constructed a bipartite network using spearman correlation (Figure 4B). This network revealed two distinct bacterial response patterns to drought. The first pattern involves common bacterial stress responses, including the PTS, Drug efflux transporter/pump, and two-component system, exemplified by *Lactobacillus intestinalis*. The second pattern is characterized by metabolic adaptations to drought, with *Cellulosilyticum* serving as a representative example. Most taxa exhibited both response patterns. Within this network, Enterobacteriaceae and *Bifidobacterium dentium* emerged as central hubs, connected to all pathways enriched in the extreme drought year 2022. For Enterobacteriaceae, the Pertussis pathway showed the strongest correlation (*ρ* = 0.95, *p* = 0.000), followed by ascorbate and aldarate metabolism (*ρ* = 0.90, *p* = 0.000), drug efflux transporter/pump (*ρ* = 0.89, *p* = 0.000), and the two-component system (*ρ* = 0.88, *p* = 0.000). For *Bifidobacterium dentium*, the strongest correlation was with the PTS pathway (*ρ* = 0.68, *p* < 0.001), followed by Carbohydrate digestion and absorption (*ρ* = 0.58, *p* < 0.001).

### 3.4 Prevalence and variability of potentially pathogenic genera in wintering common cranes over different timescales

**Figure 5:**
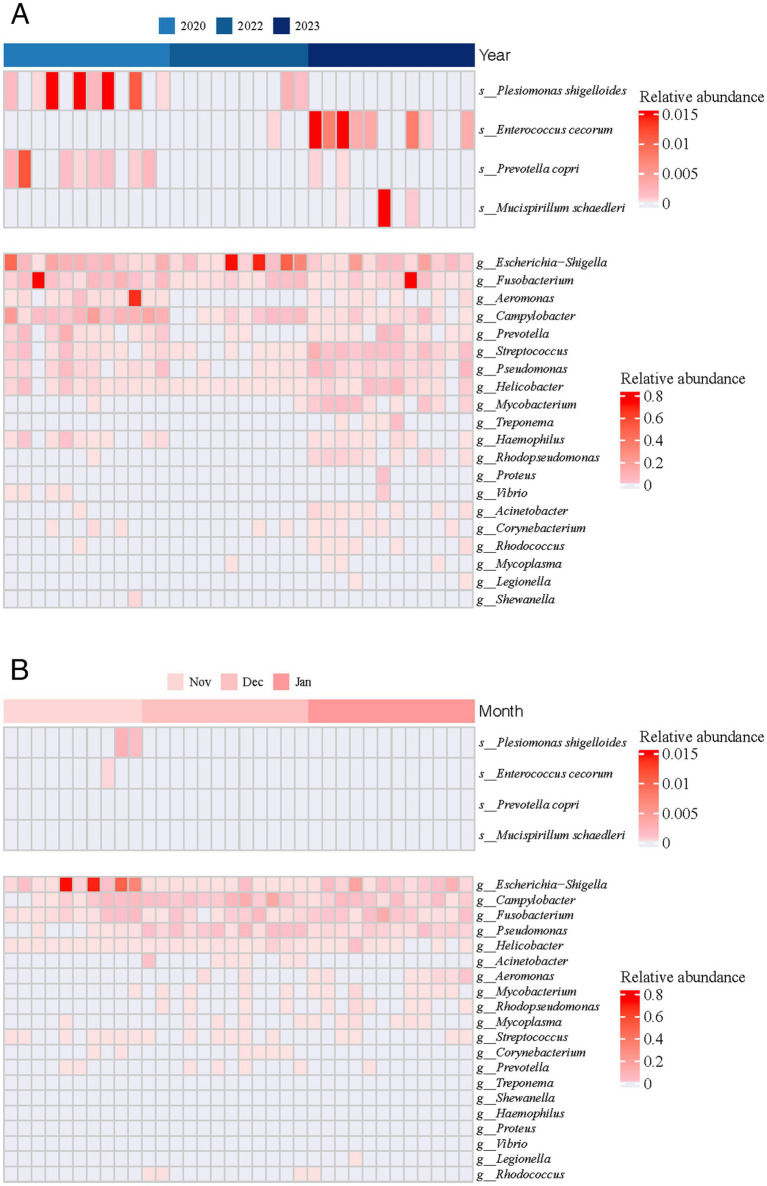
Relative abundance of 20 known potential pathogenic bacteria across both inter-annual **(A)** and inter-monthly **(B)** temporal scales.

In all fecal samples collected from wintering common cranes at Poyang Lake, we detected 20 known potentially pathogenic genera (Figure 5). Six of these genera were prevalent in over half of the samples, specifically *Escherichia-Shigella* (8.57% ± 18.43%), *Pseudomonas* (0.84% ± 1.33%), *Fusobacterium* (4.14% ± 14.80%), *Streptococcus* (0.54% ± 1.53%), *Helicobacter* (0.39% ± 1.13%), and *Campylobacter* (3.35% ± 5.60%). Among these, *Escherichia-Shigella*, *Fusobacterium*, and *Campylobacter* emerged as the primary potential pathogenic genera, each exhibiting an average relative abundance exceeding 2%. Additionally, four pathogenic species were identified, namely *Prevotella copri*, *Plesiomonas shigelloides*, *Mucispirillum schaedleri*, and *Enterococcus cecorum*. Overall, the relative abundance and occurrence frequency of these potential pathogens were generally low during the wintering period, with notably higher values recorded only in 2020.

To understand the impact of extreme drought on gut pathogenic bacteria, we conducted a comparative analysis of pathogenic genera across different periods. On an annual scale, we observed 15, 9, and 18 potentially pathogenic genera in 2020, 2022, and 2023, respectively. In 2022, the main potentially pathogenic genera were *Escherichia-Shigella* (22.75% ± 30.34%), *Campylobacter* (1.07% ± 1.59%), *Fusobacterium* (0.77% ± 1.21%), *Pseudomonas* (0.04% ± 0.04%), *Helicobacter* (0.03% ± 0.01%), and *Streptococcus* (0.02% ± 0.02%). Additionally, we noted that the relative abundances of *Pseudomonas* (2020: *p* = 0.017; 2023: *p* = 0.001), *Helicobacter* (2020: *p* = 0.004; 2023: *p* = 0.003), and *Streptococcus* (2020: *p* = 0.012; 2023: *p* < 0.001) were significantly lower in 2022 compared with other years. On a monthly scale, we identified 9, 13, and 12 potentially pathogenic genera in November, December, and January, respectively. We found that the relative abundance of *Escherichia-Shigella* in December (Nov: *p* = 0.002; Jan: *p* = 0.002) was significantly lower than in other months, and the relative abundance of *Pseudomonas* (Dec: *p* < 0.001; Jan: *p* < 0.001) in November was significantly lower than in other months.

## 4 Discussion

Gut microbiota plays a crucial role in the adaptation and survival of migratory birds by influencing their ability to respond to environmental changes (Kohl, 2012). Extreme environments, such as drought, can significantly impact the composition, stability, and function of gut microbiota by altering the birds’ habitats and food sources, thereby affecting the birds’ adaptability to changing conditions (Williams et al., 2023; Fan et al., 2022). The rapidly fluctuating hydrological conditions at Poyang Lake, driven by climatic extremes and human activities, provide a unique opportunity to investigate how the gut microbiota of wintering birds adapt to environmental stressors. In this study, we employed 16S rRNA high-throughput sequencing to examine the gut microbiota of common cranes wintering at Poyang Lake over various temporal scales, focusing on responses to extreme drought conditions. Our analysis revealed significant temporal dynamics in the gut microbiota of the wintering common cranes. Both large- (inter-annual) and small-scale (inter-monthly) temporal changes profoundly influenced gut microbiota composition and structure, with larger temporal scales exerting a more substantial impact. Extreme drought events notably shaped the diversity, composition, and functional capabilities of gut microbiota, underscoring the microbiota’s potential role in helping common cranes adapt to climatic stresses. These findings highlight the critical role of gut microbiota as an adaptive mechanism in response to environmental challenges.

Poyang Lake serves as a crucial habitat and food source for wintering migratory birds. However, these resources are highly sensitive to water-level fluctuations (Xia et al., 2010). In 2022, water levels consistently remained below 9 m during the dry season, leading to extreme drought conditions. Our study on the common crane population revealed a clear synchronization between changes in gut microbiota diversity and water level fluctuations. Specifically, the alpha diversity of the gut microbiota in common cranes was at its lowest in 2022 compared with other years. The extreme drought in 2022 resulted in the complete die-off of submerged vegetation before it could mature, creating a severe scarcity of habitat and food for the wintering migratory birds (Hu, 2023). While many migratory birds, particularly common cranes, resorted to foraging in farmlands, the drought also resulted in significant crop failures in the Poyang Lake plain. Reports indicated large-scale rice crop failures, with many grains being empty and insufficient to provide sufficient food for the migratory birds (Lei et al., 2023). Consequently, the reduced food diversity forced migratory birds to consume the few available plants and even lower-quality food such as plant roots and stems with high cellulose content, as plants aged rapidly due to the drought. This observation aligns with previous studies linking diet diversity to gut microbiome diversity, where low diet diversity correlates with low microbial diversity (Williams et al., 2023; Greene et al., 2018). Over the months, the alpha diversity of gut microbiota in common cranes gradually increased, coinciding with rising water levels. Relatively higher water levels can submerge more areas, promoting the growth of aquatic plants such as submerged macrophytes (Hou et al., 2021). These plants are a primary food source for cranes, providing essential nutrients during their wintering period. This further underscores the impact of water levels on the gut microbial diversity of wintering migratory birds by influencing their food and habitat availability.

The beta diversity of the gut microbiota in common cranes exhibited three distinct clusters at both inter-annual and-monthly temporal scales, aligning with previous research on the temporal dynamics of wintering bird microbiota (Li C. et al., 2021; Wang W. X. et al., 2021; Zhao et al., 2021). Environmental factors, particularly diet, are pivotal in shaping the gut microbiota of wild birds over time (Grond et al., 2018; Waite and Taylor, 2014). During different wintering periods, water levels at Poyang Lake significantly affect the habitat area and food resources available to migratory birds (Xia et al., 2010). On the inter-annual temporal scale, the average monthly water levels in November for the years 2020, 2022, and 2023 were 12.20 ± 1.82 m, 6.74 ± 0.24 m, and 10.27 ± 0.74 m, respectively. According to Xia et al. (2010), the most extensive suitable wintering habitat at Poyang Lake typically occurs in November when water levels are approximately 10.79 m. Both excessively high and low water levels during this period can drastically affect the availability and spatial distribution of local food resources, subsequently influencing the microbiota structure of common cranes (Li et al., 2019). On a small temporal scale, the average monthly water levels for November, December, and January were 6.74 ± 0.24 m, 7.58 ± 0.58 m, and 7.05 ± 0.19 m, respectively. The smaller water-level fluctuations between these months, relative to larger inter-annual variations, correlate with our findings that microbial community distances were significantly lower at short temporal scales than those at long temporal scales. Beyond water levels, the gradual arrival of other migratory birds as the wintering season progresses intensifies competition for food resources (Zhang et al., 2022). Additionally, potential mold growth in some grain seeds may alter the abundance and quality of food available to common cranes. These factors likely contribute to the significant differences observed in the gut microbiota structure of common cranes. These insights underscore the dynamic nature of gut microbiota in response to environmental stressors and highlight the importance of considering both temporal and ecological factors to understand the gut microbiota of migratory birds.

The gut microbiota of common cranes wintering at Poyang Lake predominantly comprises of Proteobacteria and Firmicutes. These findings are consistent with studies on various herbivorous bird species, such as the whooper swan (*Cygnus cygnus*) (Wang W. X. et al., 2021), domestic goose (*Anser anser*) (Fu et al., 2020), black-necked crane (*Grus nigricollis*) (Zhao et al., 2021), and great bustard (*Otis tarda*) (Lu et al., 2024). Proteobacteria are essential for energy accumulation, enabling cranes to acquire additional energy to withstand the cold winter (Chevalier et al., 2015). Firmicutes, on the other hand, are capable of breaking down complex sugars, polysaccharides, and fatty acids in food, aiding in the digestion of complex polymers such as polysaccharides and cellulose found in plant-based diets, thereby producing energy and nutrients for the host to absorb and utilize (Grond et al., 2018). Upon arrival at their wintering grounds, the higher proportion of Proteobacteria and Firmicutes facilitates effective digestion and increased energy absorption, allowing the cranes to adapt more swiftly to their wintering environment.

The primary food sources for wintering common cranes at Poyang Lake consist of rice and *Polygonum* species, whose biomass is significantly influenced by hydrological conditions (Hou et al., 2021). Extreme drought conditions not only reduce rice yields around Poyang Lake but also compel common cranes to consume lower-quality food, such as the roots and stems of *Polygonum* species, which contain high cellulose content and age rapidly due to the drought (Hu, 2023). During the extreme drought year of 2022, there was an increased enrichment of Enterobacteriaceae, a family within the Proteobacteria phylum. These bacteria are involved in glucose fermentation and nitrate reduction to nitrite, processes that can assist common cranes in adapting to drought-induced stress (Rock and Donnenberg, 2014). Additionally, the genus *Cellulosilyticum*, known for its ability to break down cellulose and convert plant polysaccharides into simpler molecules such as glucose, exhibited a significant rise in abundance. These molecules can be further fermented into short-chain fatty acids, which are crucial for herbivores that rely on plant-based diets (Gao et al., 2024). Furthermore, Bifidobacteriaceae, along with other anaerobic bacteria, form a protective biofilm on the gut mucosa, ensuring gut microbiota stability and maintaining gut function balance (Becker et al., 2014). In 2022, we observed a significant increase in the relative abundance of Enterobacteriaceae, *Cellulosilyticum*, and two species from the Bifidobacteriaceae family relative to the other years, with their abundance fluctuations closely mirroring changes in water levels. This observation aligns with previous research indicating that gut microbiota help the host adapt to environmental changes (Kokou et al., 2018; Huo et al., 2020). Additionally, the arrival of other migratory birds during the wintering season can intensify competition for food resources, further affecting the gut microbiota composition of common cranes.

Based on the predicted gene functions derived from the 16S rRNA sequences of the gut microbiota in common cranes, KEGG level 2 functions were predominantly associated with carbohydrate metabolism, membrane transport, amino acid metabolism, cofactor and vitamin metabolism, and energy metabolism. These findings are consistent with those observed in other bird species (Fu et al., 2020; Zhao et al., 2021). In 2022, the functional capabilities of the cranes’ gut microbiota exhibited significant changes, with notable enrichment in carbohydrate metabolism, the PTS, and the two-component system. The PTS is primarily involved in phosphorylating various sugars and their derivatives through a phosphotransfer mechanism, facilitating their transport into the cell. Additionally, it plays a role in regulating virulence in certain pathogens and mediating stress responses (Liu et al., 2020). The two-component system, a prevalent bacterial signaling mechanism, participates in the regulation of antibiotic resistance and biofilm formation (Yu et al., 2020). These shifts may partly account for the observed reduction in the relative abundance of pathogenic bacteria under extreme drought conditions. The enrichment of these functional genes was most strongly associated with the Enterobacteriaceae family, presumably due to the host experiencing stress. Combined with bipartite network analysis, these findings suggest that the gut microbiota of common cranes adapts to extreme drought by enhancing its carbohydrate metabolism and stress response regulation capacity. Such adaptive changes likely help the cranes better cope with challenging environmental conditions, underscoring the dynamic nature of gut microbiota in response to severe ecological stressors.

We detected various potential pathogenic bacteria in the feces of wintering common cranes at Poyang Lake, with *Escherichia-Shigella* being particularly abundant. This genus, belonging to the phylum Proteobacteria, is a highly infectious intestinal pathogen capable of invading and causing severe inflammation and tissue damage in the colorectal epithelium (Carayol and Nhieu, 2013). Through biochemical and genetic methods, Oaks et al. (2010) observed that *Escherichia* may cause the death of Alaska redpoll finches (*Carduelis flammea*) and identified this type of bacteria in other birds that had died or were subclinically infected. The relative abundance of these potential pathogens varied over time, and most could not be identified at the species level. During the extreme drought year of 2022, the diversity of potential pathogens was notably low, coinciding with the lowest microbial diversity observed. This finding contrasts with numerous studies reporting a negative correlation between gut microbiota diversity and pathogen diversity (Guan et al., 2016; de Vos and de Vos, 2012; Manichanh et al., 2006).

The area surrounding Poyang Lake is dotted with numerous poultry farms, where local farmers traditionally raise poultry in rice fields to feed on leftover grains (Choi et al., 2016). In recent years, common cranes have increasingly shifted their foraging grounds from lakeshore grasslands to these rice fields (Jiang et al., 2015). This enhanced interaction between wild birds, poultry, and humans increases the likelihood of gut microbiota transmission among different hosts. Such interactions may contribute to the observed fluctuations in the relative abundance of pathogens in the gut microbiota of common cranes over different time scales. Additionally, the large congregations of waterbirds in farmland areas, driven by the degradation of submerged vegetation, further exacerbate this issue. As natural wetlands degrade, tens of thousands of waterbirds, including cranes and geese, are forced to forage in artificial wetlands (Hou et al., 2021). These large congregations can enhance the transmission of pathogens between species, leading to changes in the relative abundance of pathogens in the gut microbiota of common cranes. While the guts of wild birds can harbor various potential pathogens (Grond et al., 2018), monitoring their gut microbiota offers a valuable approach to detecting invasive pathogens. This can significantly reduce the risk of disease transmission within and between species, thereby preventing the outbreak and spread of zoonotic diseases (Fu et al., 2020; Gao et al., 2021; Wei et al., 2019).

It is important to acknowledge several limitations in our study. Firstly, our analysis on the impact of drought on the gut microbiota of birds at Poyang Lake was limited to observations made during the first month after the arrival of common cranes for each year. This narrow scope did not account for variations across different months, potentially overlooking inter-monthly fluctuations. Additionally, our study lacked comprehensive data on the diet composition of common cranes as well as the year-to-year variation in available food resources. Consequently, the hypothesis that drought conditions lead to alterations in the dietary intake of common cranes, subsequently influencing the structure and function of their gut microbiota, remains largely speculative. To rigorously test this hypothesis, further experimental studies are necessary. These studies should incorporate a broader temporal scope and include detailed dietary analyses, which would significantly enhance the rigor and validity of our findings. Moreover, integrating longitudinal studies that monitor these variables over multiple years could provide deeper insights into the long-term effects of environmental changes on avian gut microbiota.

## 5 Conclusion

Our study investigates the temporal dynamics of gut microbiota composition and structure in common cranes wintering at Poyang Lake across inter-annual and-monthly temporal scales. The findings highlight the resilience and adaptability of the gut microbiota in response to environmental changes, particularly under extreme drought conditions. The maintenance of a core microbiota composition, alongside the fluctuation in specific microbial groups and functions, illustrates the complex interplay between gut microbiota and environmental factors. This research enhances our understanding of gut microbiota dynamics in migratory birds and underscores the importance of comprehensive studies to investigate the impacts of climatic changes on wildlife health and conservation.

## Data Availability

The data presented in the study are deposited in the NCBI repository, accession number PRJNA1183331.

## Footnotes

^1^http://slt.jiangxi.gov.cn/col/col28224/index.html
