# Supplementary figures and images for: Extreme drought shapes the gut microbiota composition and function of common cranes (Grus grus) wintering in Poyang Lake
Source: Front Microbiol. 2024 Nov 20;15:1489906. doi: 10.3389/fmicb.2024.1489906 (PMC11614848; doi:10.3389/fmicb.2024.1489906)

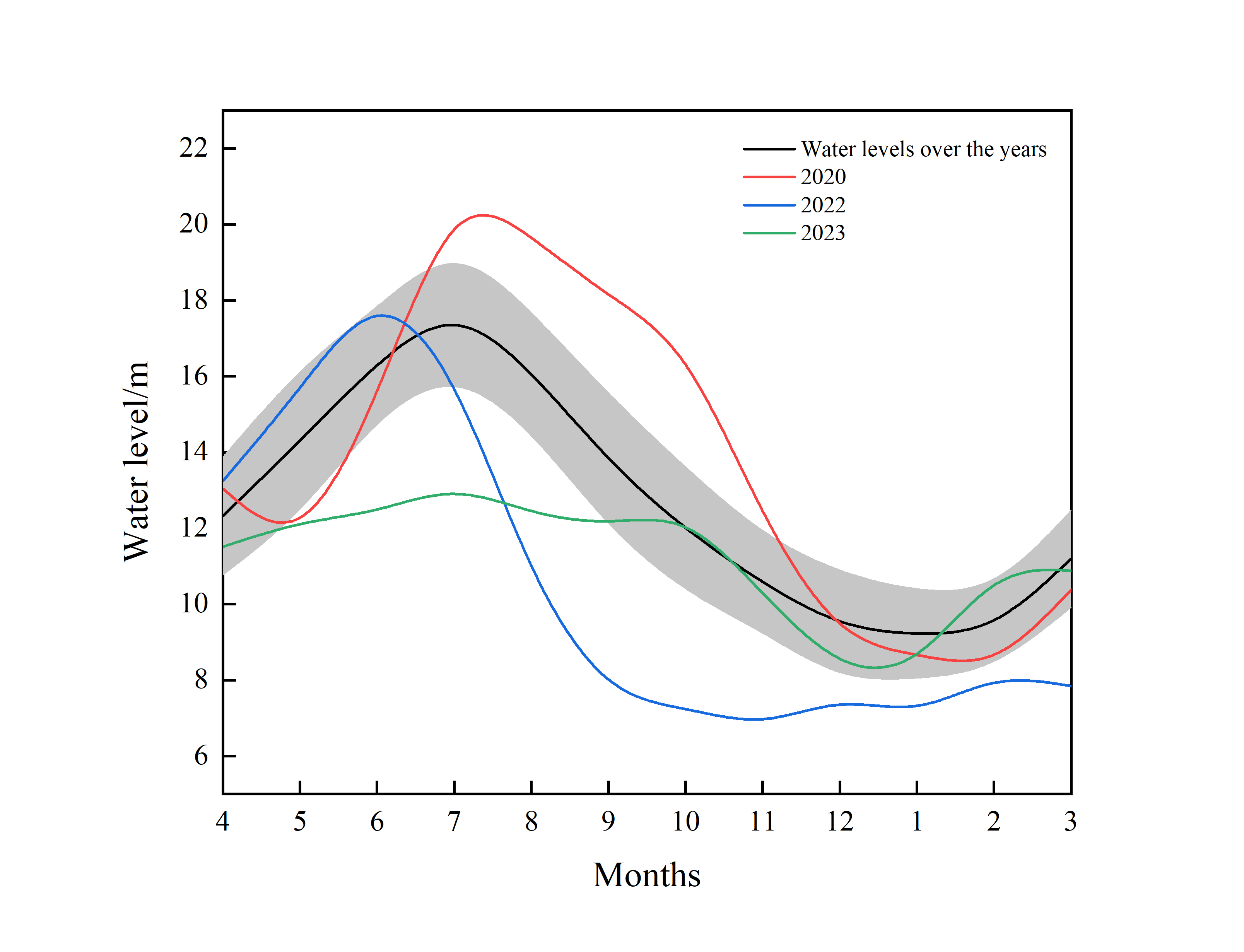

Supplement: Supplementary file 1 [file Image_1.PNG]

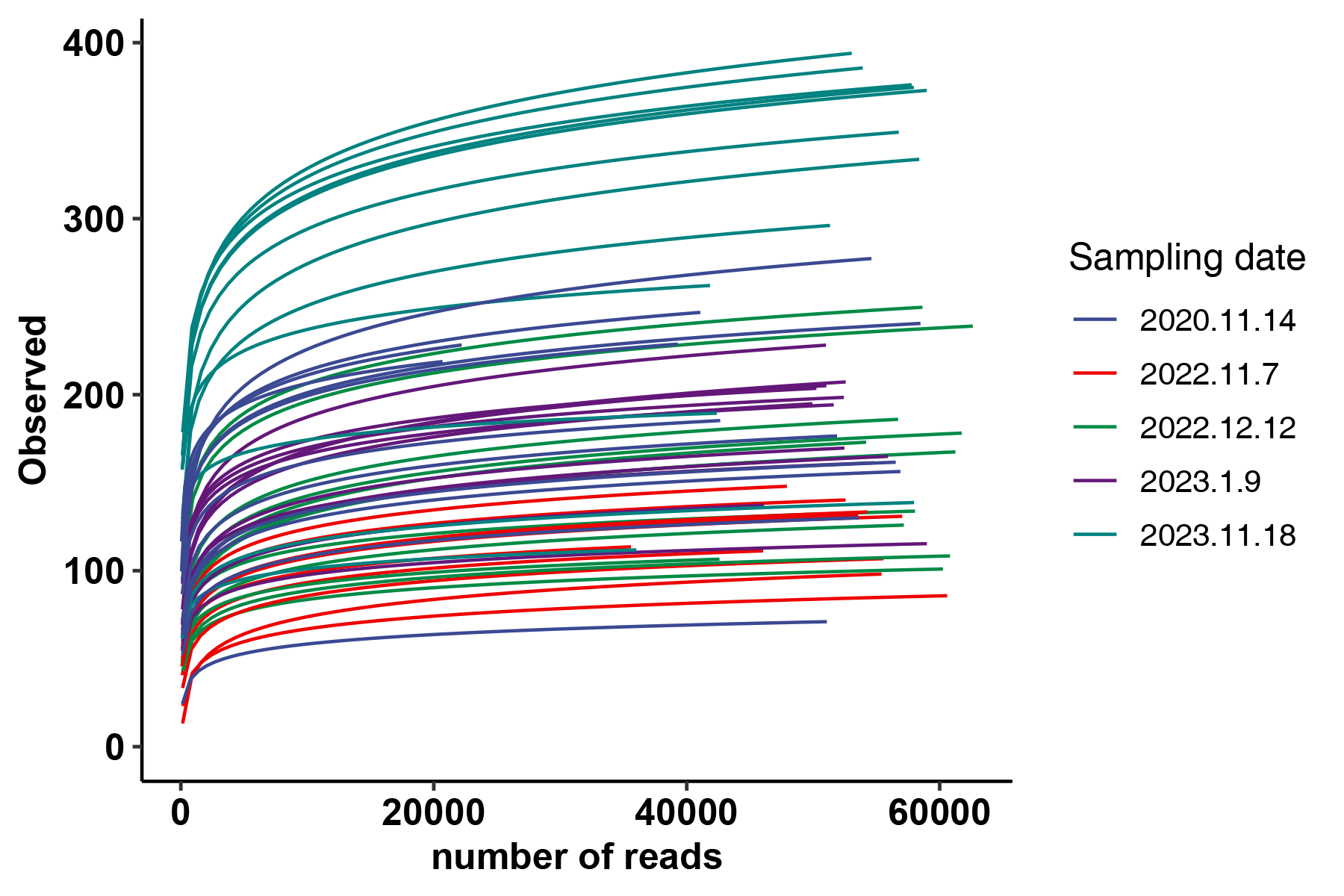

Supplement: Supplementary file 2 [file Image_2.TIF]

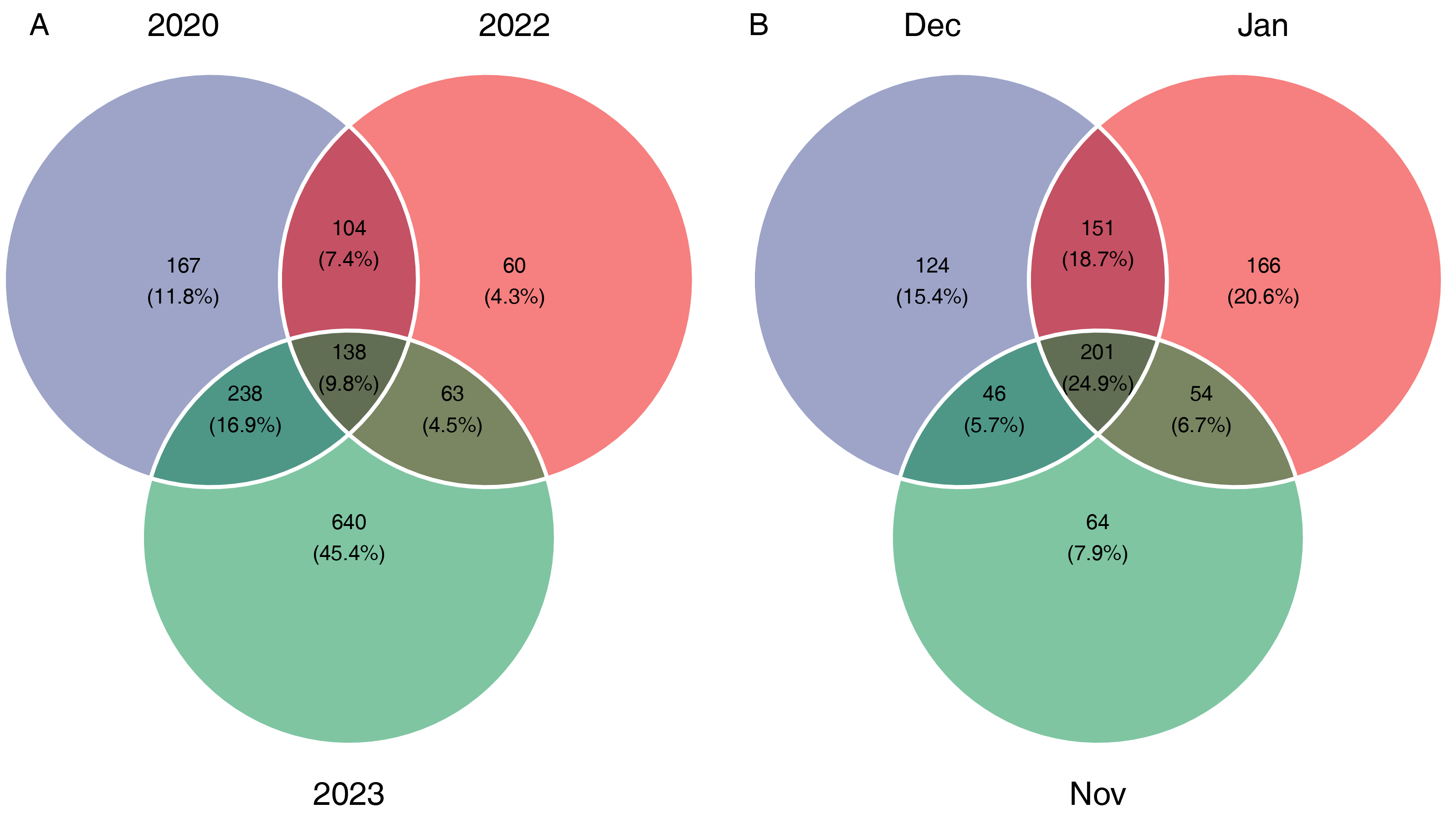

Supplement: Supplementary file 3 [file Image_3.TIF]

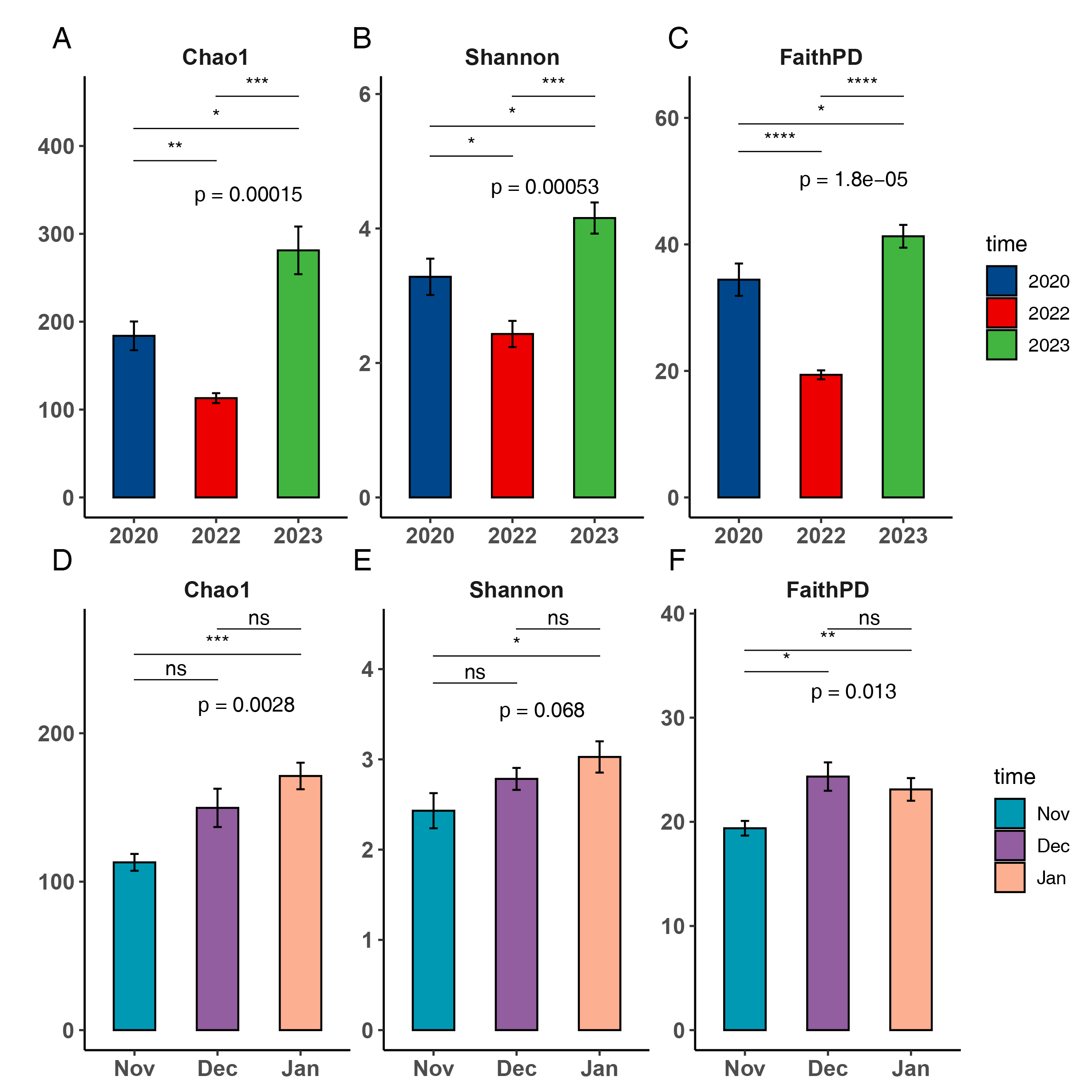

Supplement: Supplementary file 4 [file Image_4.TIF]

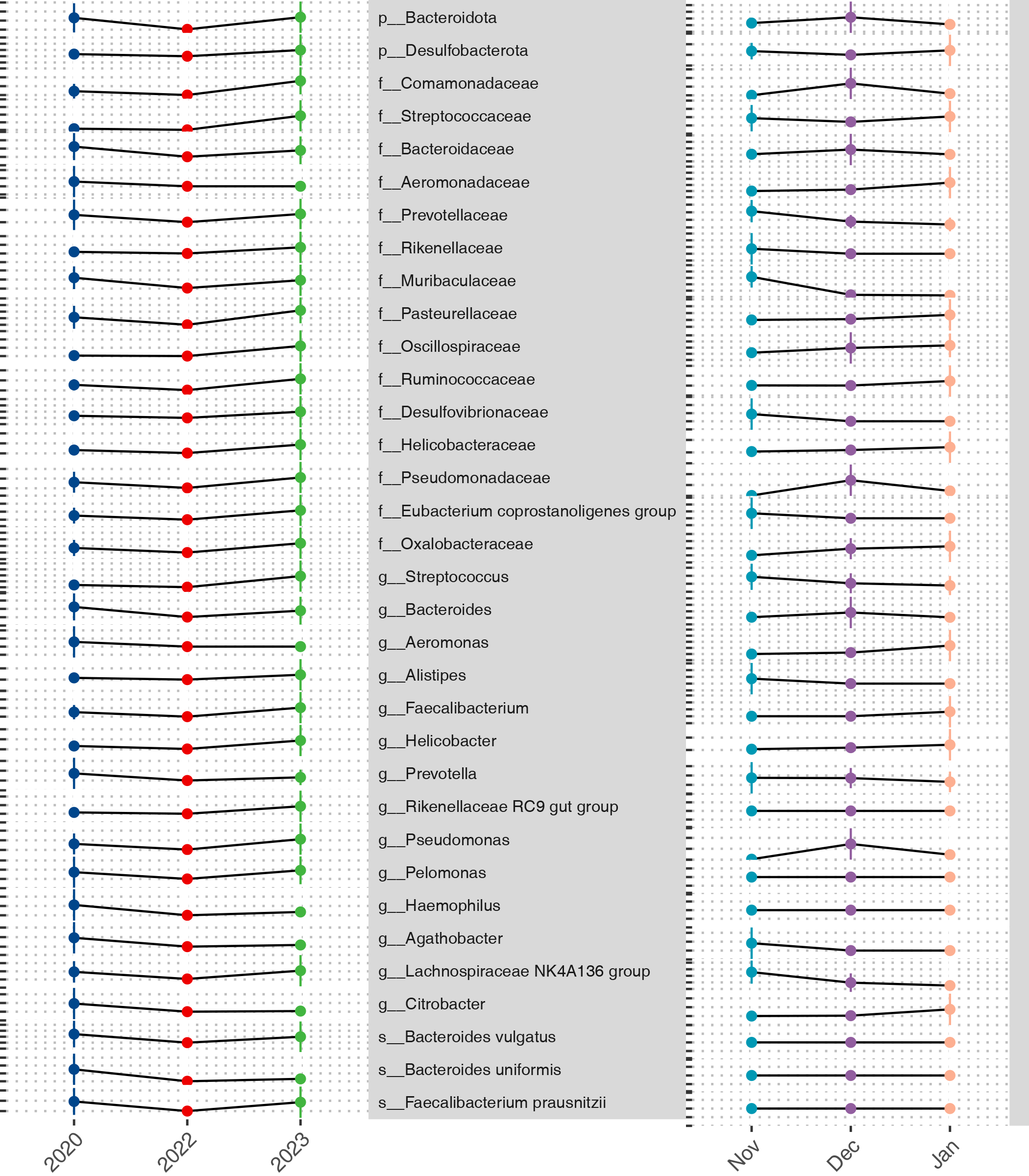

Supplement: Supplementary file 5 [file Image_5.TIF]

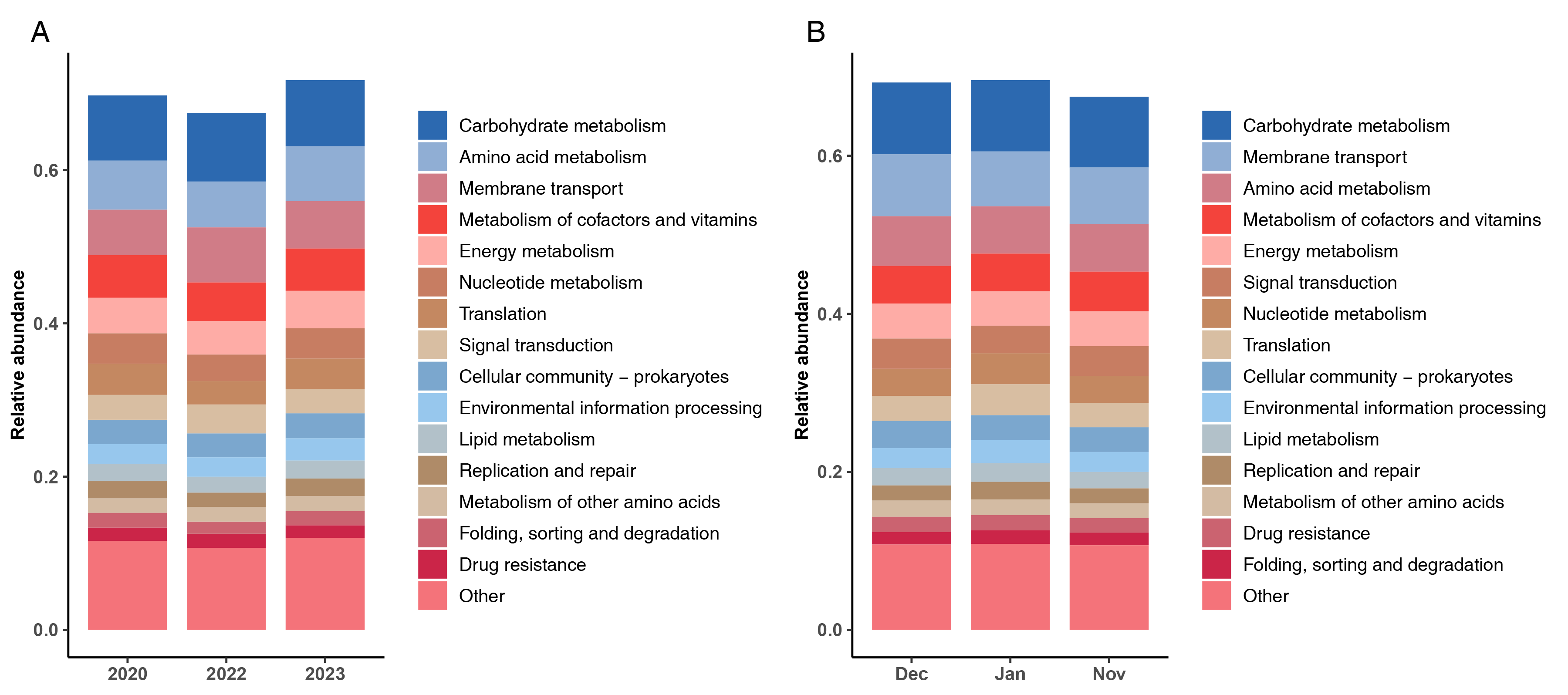

Supplement: Supplementary file 6 [file Image_6.TIF]

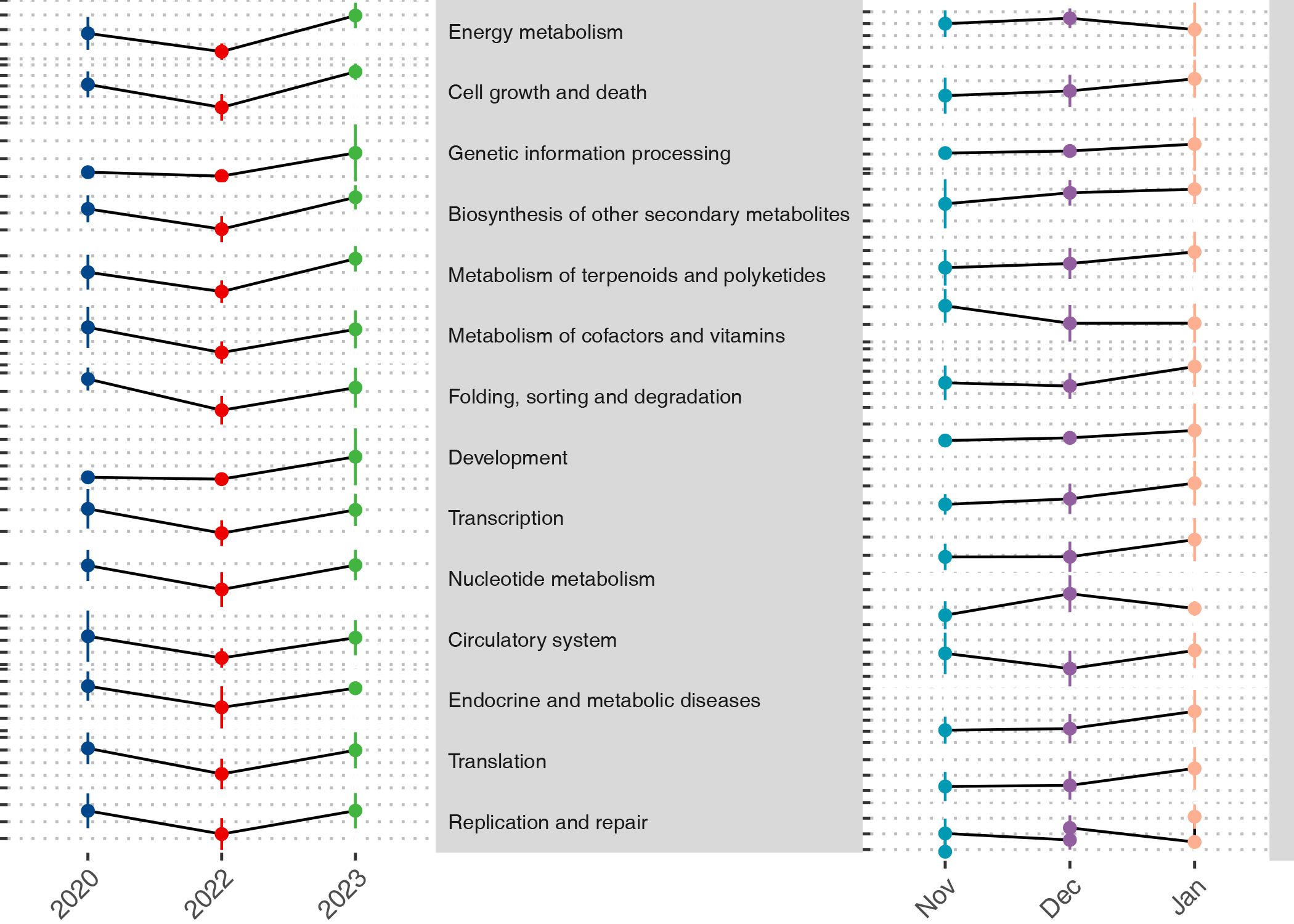

Supplement: Supplementary file 7 [file Image_7.TIF]
