# Supplementary material for: Extreme drought shapes the gut microbiota composition and function of common cranes (Grus grus) wintering in Poyang Lake
Source: Front Microbiol. 2024 Nov 20;15:1489906. doi: 10.3389/fmicb.2024.1489906 (PMC11614848; doi:10.3389/fmicb.2024.1489906)
Supplement: Supplementary file 8 [file Table_1.docx]

**Supplementary Table Legends**

**Table S1** Average relative abundance of the top 10 bacterial phyla in Common Crane samples.

**Table S1**

| Phylum | 2020 | 2022/Nov | Dec | Jan | 2023 | Total |
| --- | --- | --- | --- | --- | --- | --- |
| Proteobacteria | 31.29% | 55.39% | 62.65% | 34.71% | 30.39% | 42.45±25.94% |
| Firmicutes | 36.48% | 42.11% | 31.06% | 59.93% | 34.57% | 40.79±22.54% |
| Bacteroidota | 13.03% | 0.31% | 1.18% | 0.11% | 13.75% | 5.86±12.21% |
| Fusobacteriota | 9.85% | 0.77% | 0.65% | 1.71% | 8.14% | 4.34±13.56% |
| Campilobacterota | 8.22% | 1.10% | 3.66% | 1.53% | 1.36% | 3.25±4.91% |
| Actinobacteriota | 0.58% | 0.32% | 0.77% | 1.84% | 9.58% | 2.70±6.26% |
| Desulfobacterota | 0.34% | 0.00% | 0.00% | 0.00% | 0.99% | 0.28±1.19% |
| Cyanobacteria | 0.00% | 0.00% | 0.04% | 0.05% | 0.61% | 0.15±0.71% |
| Spirochaetota | 0.00% | 0.00% | 0.00% | 0.00% | 0.24% | 0.05±0.36% |
| Deferribacterota | 0.06% | 0.00% | 0.00% | 0.00% | 0.15% | 0.04±0.23% |
