# Supplementary material for: Extreme drought shapes the gut microbiota composition and function of common cranes (Grus grus) wintering in Poyang Lake
Source: Front Microbiol. 2024 Nov 20;15:1489906. doi: 10.3389/fmicb.2024.1489906 (PMC11614848; doi:10.3389/fmicb.2024.1489906)
Supplement: Supplementary file 9 [file Table_2.docx]

**Supplementary Table Legends**

**Table S2** Average relative abundance of bacterial genera with relative abundance exceeding 2% in Common Crane samples.

**Table S2**

| Genus | 2020 | 2022/Nov | Dec | Jan | 2023 | Total |
| --- | --- | --- | --- | --- | --- | --- |
| *Enterobacter* | 2.62% | 28.13% | 35.68% | 15.61% | 6.20% | 19.00±24.00% |
| *Lactobacillus* | 6.52% | 2.32% | 14.14% | 25.96% | 3.83% | 11.59±16.16% |
| *Escherichia-Shigella* | 8.83% | 22.75% | 0.27% | 2.96% | 4.52% | 8.57±18.43% |
| *Clostridium sensu stricto 1* | 4.00% | 11.03% | 1.25% | 7.76% | 1.98% | 5.28±8.25% |
| *Catellicoccus* | 3.11% | 0.92% | 12.50% | 5.59% | 0.02% | 4.96±11.01% |
| *Terrisporobacter* | 3.38% | 8.92% | 1.36% | 7.08% | 1.49% | 4.52±9.20% |
| *Fusobacterium* | 8.18% | 0.77% | 0.65% | 1.67% | 6.48% | 4.14±14.80% |
| *Campylobacter* | 7.89% | 1.07% | 3.56% | 1.31% | 0.49% | 3.35±5.60% |
| *Romboutsia* | 3.68% | 2.37% | 0.56% | 5.77% | 1.76% | 3.10±4.23% |
| *Klebsiella* | 0.91% | 0.65% | 8.68% | 0.95% | 0.92% | 2.67±7.23% |
| *Bacteroides* | 6.18% | 0.10% | 0.82% | 0.07% | 3.91% | 2.62±6.46% |
| *Kosakonia* | 0.53% | 0.03% | 3.05% | 5.25% | 0.53% | 2.11±6.90% |
